# Characterization of a Ran gene from *Puccinia striiformis* f. sp. *tritici* involved in fungal growth and anti-cell death

**DOI:** 10.1038/srep35248

**Published:** 2016-10-13

**Authors:** Yulin Cheng, Juanni Yao, Yanru Zhang, Shumin Li, Zhensheng Kang

**Affiliations:** 1 State Key Laboratory of Crop Stress Biology for Arid Areas, College of Life Sciences, Northwest A&F University, Yangling 712100, Shaanxi, China; 2 State Key Laboratory of Crop Stress Biology for Arid Areas, College of Plant Protection, Northwest A&F University, Yangling 712100, Shaanxi, China

## Abstract

Ran, an important family of small GTP-binding proteins, has been shown to regulate a variety of important cellular processes in many eukaryotes. However, little is known about Ran function in pathogenic fungi. In this study, we report the identification and functional analysis of a Ran gene (designated *PsRan*) from *Puccinia striiformis* f. sp. *tritici (Pst*), an important fungal pathogen affecting wheat production worldwide. The PsRan protein contains all conserved domains of Ran GTPases and shares more than 70% identity with Ran proteins from other organisms, indicating that Ran proteins are conserved in different organisms. *PsRan* shows a low level of intra-species polymorphism and is localized to the nucleus. qRT-PCR analysis showed that transcript level of *PsRan* was induced *in planta* during *Pst* infection. Silencing of *PsRan* did not alter *Pst* virulence phenotype but impeded fungal growth of *Pst*. In addition, heterologous overexpression of *PsRan* in plant failed to induce cell death but suppressed cell death triggered by a mouse BAX gene or a *Pst* Ras gene. Our results suggest that *PsRan* is involved in the regulation of fungal growth and anti-cell death, which provides significant insight into Ran function in pathogenic fungi.

Small GTP-binding proteins in eukaryotes from yeast to human constitute a superfamily, which includes more than 100 members and is structurally classified into at least five families: Ras, Rho, Rab, Sar1/Arf, and Ran^1^. The Ran (Ras-related nuclear) protein was originally isolated as a homolog of Ras proteins and eukaryotes usually contain one to four Ran genes^2^. As the only known family of small GTP-binding proteins primarily localized inside the nucleus, Ran is originally thought to be devoted to the nuclear translocation of proteins^3^,^4^. However, Ran is now known to expand its important influence to nuclear assembly, mRNA processing, and cell cycle control^5^,^6^. Recent researches indicate that Ran also plays an important role in human cancer^7^ and apoptotic cell death^8^, animal immunity against virus infection^9^, animal development and reproduction^10^, and plant development and mediated responses to the environment^11^,^12^. Increased evidences suggest that Ran is involved in the regulation of a variety of important cellular processes in different eukaryotes.

As with other living organisms, pathogenic fungi that are the causes of deadly diseases in human, animals, and plants use numerous signal-transduction systems to sense and respond to their environments^13^. Small GTP-binding proteins are molecular switches in cellular signal transduction pathways^14^, and many members of the four families (Ras, Rho, Rab, and Sar1/Arf) in pathogenic fungi were proven to regulate a variety of important biological processes^15^,^16^,^17^,^18^. Noticeably, Ras proteins, the most well-known family of small GTP-binding proteins, act upstream of mitogen-activated protein kinase (MAPK) or cyclic adenosine monophosphate-protein kinase A (cAMP-PKA) pathways and appear to play important roles in fungal growth, asexual and sexual reproduction, virulence, and cell death of pathogenic fungi^19^,^20^,^21^,^22^. Some genes encoding putative fungal Ran proteins were identified in several pathogenic fungi (*Fusarium graminearum*, *Colletotrichum acutatum*, *Puccinia striiformis* f. sp. *tritici*, and *Beauveria bassiana*)^23^,^24^,^25^,^26^, but little is currently known about Ran function in pathogenic fungi. Thus, the identification and functional analysis of Ran genes from pathogenic fungi will lead to better understanding of their specific roles in pathogenic fungi.

As an important plant pathogenic fungus, *Puccinia striiformis* f. sp. *tritici (Pst*) can cause the wheat stripe rust disease that is one of the most important wheat diseases worldwide. Significant wheat yield losses caused by outbreaks of stripe rust have resulted in economic losses throughout human history^27^. Thus, the understanding of *Pst* pathogenesis and searching for novel pathogen control strategies are of great significance to durably control the wheat stripe rust disease. As an obligate biotroph pathogen, *Pst* grows only *in planta* and lacks an efficient and reliable system for stable transformation, which has long hindered the study of putative pathogenic genes. Recently, host-induced gene silencing (HIGS) has been developed and has proven to be a useful tool to study genes in obligate biotrophic pathogens^28^,^29^. Our recent study investigated the specific function of two *Pst* Ras genes using the barley stripe mosaic virus (BSMV)-mediated HIGS and heterologous expression assays, which showed that *PsRas1* and *PsRas2* are involved in rust pathogenicity and cell death, respectively^30^. The goals of the present study were to identify gene(s) encoding Ran protein(s) from *Pst* and to determine its or their specific functions. We found that *Pst* contains only one Ran gene and it plays an important role in the regulation of fungal growth and anti-cell death, which provides significant insight into Ran function in pathogenic fungi.

## Results

### Identification of one Ran gene from *Pst*

**Figure 1:**
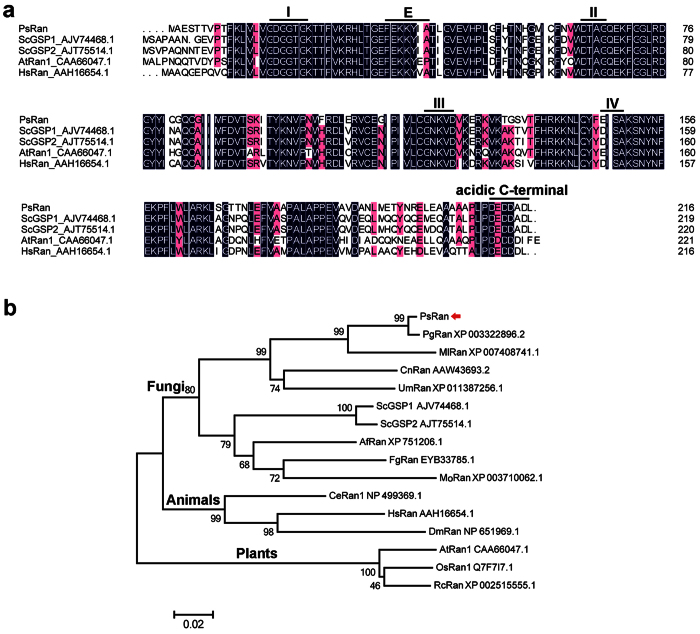
Sequence alignment and phylogenetic analysis of PsRan and other Ran proteins in different organisms. (**a**) Sequence alignment of PsRan and Ran proteins in three other organisms, including *Saccharomyces cerevisiae (Sc*), *Arabidopsis thaliana (At*) and *Homo sapiens (Hs*). Lines indicated six conserved domains for Ran GTPases, including four guanine nucleotide-binding domains (I–IV), an effector domain (E), and an acidic C-terminal domain. (**b**) Phylogenic analysis of PsRan and other Ran proteins in several fungi, animals, and plants. *P. graminis* f. sp. *tritici (Pg*), *P. triticina (Pt*), *Melampsora larici-populina (Ml*), *Cryptococcus neoformans (Cn*), *Ustilago maydis (Um*), *S. cerevisiae (Sc*), *Aspergillus fumigatus (Af*), *F. graminearum (Fg*), and *Magnaporthe oryzae (Mo*) are grouped as fungi; *Caenorhabditis elegans (Ce*), *Homo sapiens (Hs*), and *Drosophila melanogaster (Dm*) are grouped as animals; *Arabidopsis thaliana (At*), *Oryza sativa (Os*), and *Ricinus communis (Rc*) are grouped as plants. Phylogenetic analysis was carried out with the MEGA5 software by the neighbor-joining method and the red arrow indicates PsRan.

One cDNA clone (WRIC_343) encoding a putative fungal Ran protein was identified in the cDNA library of *Pst*-infected wheat leaves^26^. Mapping to *Pst* genome in the Broad Institute *Puccinia* database showed that the corresponding gene, *PSTG_13752.1* (designated *PsRan*), has an open reading frame (ORF) of 651 bp that encodes a 216 aa protein with a predicted molecular mass of 24.07 kDa. Further sequence alignment revealed that PsRan shows high similarity (more than 70%) with Ran proteins from other organisms, including *Saccharomyces cerevisiae, Arabidopsis thaliana*, and *Homo sapiens* (Fig. 1a). The PsRan protein has four guanine nucleotide-binding domains, an effector domain, and an acidic C-terminal domain, which are the characteristics of Ran GTPases^1^,^31^ and are highly conserved during evolution (Fig. 1a). These results suggest that *PsRan* is a typical Ran gene.

Because the model yeast *S. cerevisiae* contains two Ran proteins, including GSP1 and GSP2^32^, a BLAST search using GSP1 and GSP2 as queries in the Broad Institute *Puccinia* database was done to make sure whether *Pst* contains other genes encoding Ran proteins besides *PsRan*. There is no other Ran GTPase-encoding gene besides *PsRan (PSTG_13752.1*) in *Pst* genome (Supplementary Figs S1 and S2), indicating *Pst* contains only one Ran gene, which is different from *S. cerevisiae*. Other two wheat rust fungi in the Broad Institute *Puccinia* database, including *P. graminis* f. sp. *tritici (Pgt*) and *P. triticina (Pt*), also contain only one Ran GTPase-encoding gene (Supplementary Figs S1 and S2).

In addition, a phylogenetic analysis of Ran proteins from various organisms resulted into two distinct phylogenetic clades (Fig. 1b). Ran proteins in plants are grouped into a clade and Ran proteins in fungi and animals are grouped into another clade (Fig. 1b), indicating a closer relationship of Ran in fungi and animals.

### Low level of intra-species polymorphism in *PsRan*

To identify intra-species polymorphism in *PsRan*, we compared its ORF sequences in ten different *Pst* isolates, including five Chinese isolates (CYR23, CYR29, CYR31, CYR32, and Su11-4), three US isolates (PST-21, PST-43, and PST-130), and two UK isolates (PST-08-21 and PST-87-7). Only two single-nucleotide polymorphisms (SNPs) are observed for *PsRan* (Supplementary Fig. S3a). What’s more, all the two SNPs are synonymous and cannot cause the sequence change of amino acids (Supplementary Fig. S3b). Our results indicate that *PsRan* shows a low level of intra-species polymorphism and is highly conserved in different *Pst* isolates.

### Nuclear localization of PsRan

**Figure 2:**
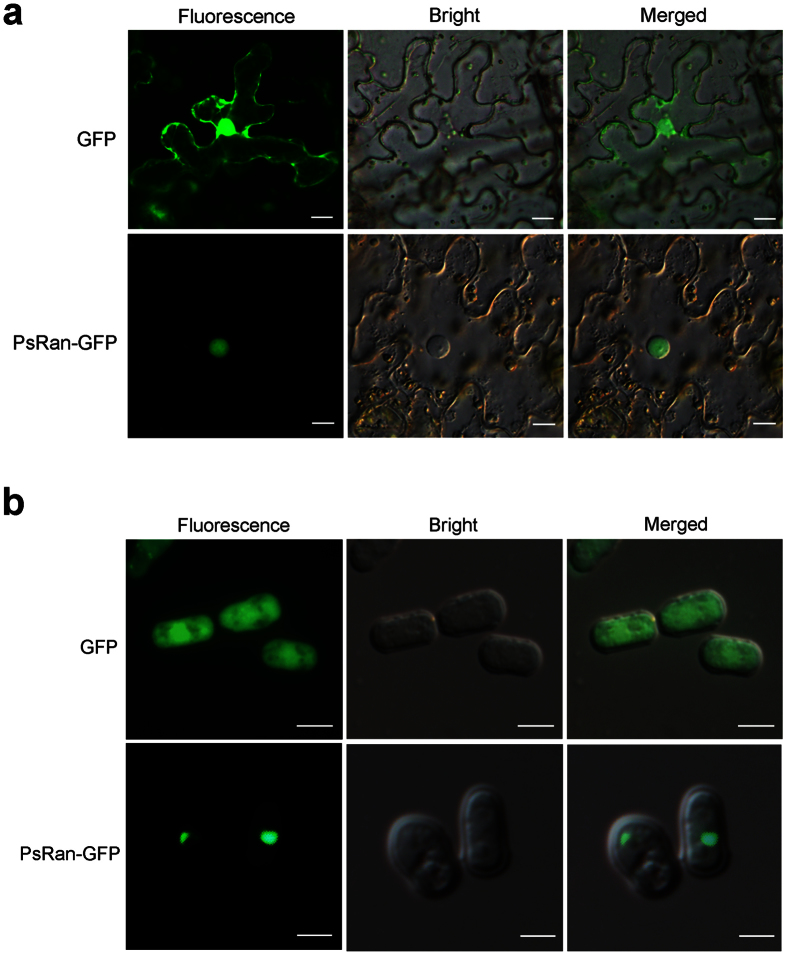
Subcellular localization of PsRan in *N. benthamiana* and *S. pombe*. (**a**) Overexpression of PsRan-GFP fusion protein and only GFP (control) in *N. benthamiana* using *A. tumefaciens*. Bar = 20 μm. (**b**) Overexpression of PsRan-GFP fusion protein and only GFP (control) in *S. pombe* using the yeast expression vector pREP3X. Bar = 5 μm.

Previous studies have shown that Ran proteins primarily localize inside the nucleus^33^,^34^. Because there is no effective transformation system for *Pst*, we conducted localization experiments of PsRan in *Nicotiana benthamiana* and fission yeast *Schizosaccharomyces pombe*, which are easier to operate as a model plant and fungus, respectively. When a PsRan-GFP fusion protein was heterologously expressed in *N. benthamiana* leaves, its fluorescence was restricted to the nucleus, while the control expressing only GFP exhibited fluorescence throughout the cell (Fig. 2a). When we transiently expressed the PsRan-GFP fusion protein in *S. pombe*, we found that its fluorescence was again restricted to the nucleus (Fig. 2b). These results indicate the nuclear localization of PsRan when using a transient and distant system.

### Transcript level of *PsRan* is induced *in planta* during *Pst* infection

**Figure 3:**
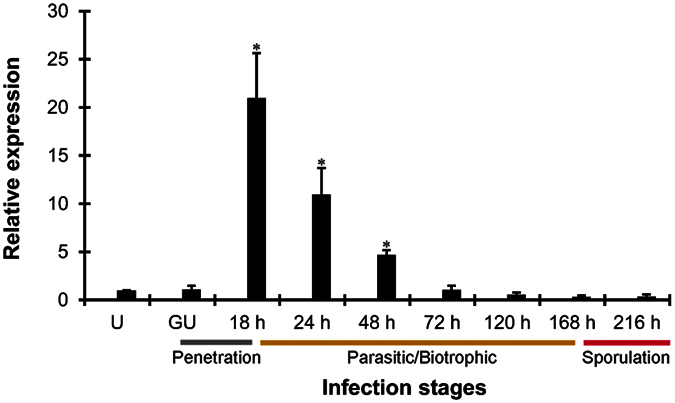
Transcription profiles of *PsRan* in different *Pst* infection stages by qRT-PCR. *Pst* development in wheat can be divided into three major stages, penetration stage (gray), parasitic/biotrophic stage (yellow), and sporulation stage (red). U, urediniospores; GU, *in vitro* germinated urediniospores; 18 h–216 h, wheat leaves infected with *Pst* at 18–216 hpi. Relative transcript levels were calculated by the comparative 2^−ΔΔCT^ method and values are expressed relative to an endogenous *Pst* reference gene *EF1*. Results are composed of the means ± standard errors of three biological replications (each done in triplicates). Asterisks indicate a significant difference (*P* < 0.05) verus uredinospores using Student’s t test.

To characterize transcript profiles of *PsRan* in different *Pst* infection stages, we assayed its transcript abundance by qRT-PCR. Compared with the transcript abundance in urediniospores, *PsRan* showed significantly increased transcript abundance in infected wheat leaves at 18, 24, and 48 hours post-inoculation (hpi), which belong to the “parasitic/biotrophic” phase of *Pst* (Fig. 3). At 18 hpi, the transcript of *PsRan* was peaked (up to 21-fold) (Fig. 3). Our results reveal that *PsRan* is an *in planta* induced gene and is preferentially expressed during the “parasitic/biotrophic” phase.

### Silencing of *PsRan* does not alter *Pst* virulence phenotype

**Figure 4:**
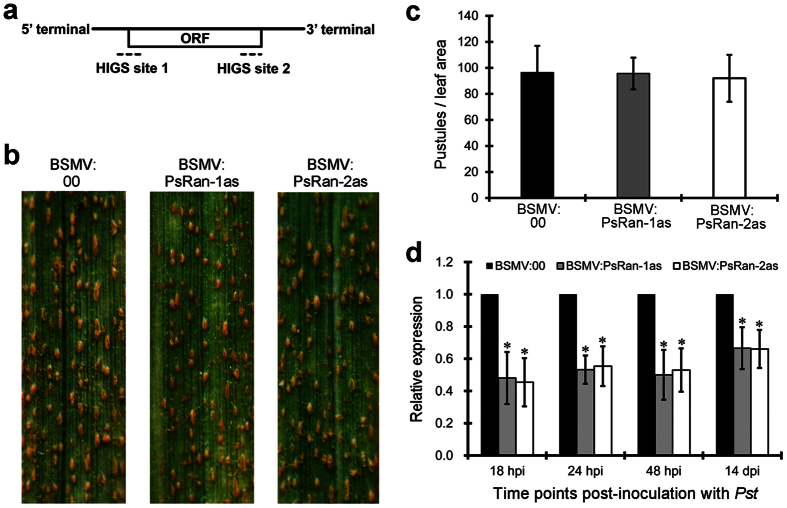
HIGS of *PsRan* did not alter *Pst* virulence phenotype. (**a**) Two specific sequence regions for HIGS of *PsRan*. (**b**) Phenotypes of the fourth leaves of BSMV:00- (control), BSMV:PsRan-1as-, and BSMV:PsRan-2as-inoculated wheat plants at 14 dpi with *Pst*. (**c**) Quantification of uredinial density in BSMV:00-, BSMV:PsRan-1as-, and BSMV:PsRan-2as-inoculated wheat plants 14 dpi with *Pst*. Values represent mean ± standard errors of three biological replications (each done in triplicates). (**d**) Relative transcript levels of *PsRan* in BSMV:00-, BSMV:PsRan-1as-, and BSMV:PsRan-2as-inoculated wheat plants at 18 hpi, 24 hpi, 48 hpi, and 14 dpi with *Pst*. Values are expressed relative to endogenous *Pst* reference gene *EF1*, with the empty vector (BSMV:00) set at 1. Values represent mean ± standard errors of three biological replications (each done in triplicates). Differences were assessed using Student’s t-tests and asterisks indicate *P* < 0.05.

To investigate the role of *PsRan* in rust pathogenicity, we silenced it by using the BSMV-mediated HIGS system. Two different fragments (PsRan-1as and PsRan-2as) (Fig. 4a) were designed for specifically silencing of *PsRan*. Ten days after inoculation with BSMV, obvious photo bleaching was observed in BSMV:TaPDSas-inoculated plants that had the wheat phytoene desaturase (PDS) gene silenced (Supplementary Fig. S4), suggesting that the RNAi system is effective. The BSMV:00- (control), BSMV:PsRan-1as-, and BSMV:PsRan-2as-inoculated wheat plants were then inoculated with virulent *Pst* isolate CYR32, and their rust disease phenotypes were photographed at 14 days post-inoculation (dpi) with *Pst*. Both BSMV:PsRan-1as- and BSMV:PsRan-2as-inoculated wheat plants showed similar disease phenotypes as control wheat plants, with equivalent amounts of uredinia as control wheat plants (Fig. 4b,c). However, qRT-PCR analysis showed that transcript level of *PsRan* was significantly reduced in BSMV:PsRan-1as- and BSMV:PsRan-2as-inoculated wheat plants compared to that in control plants (Fig. 4d), indicating *PsRan* was partially knocked down by the RNAi. These results suggest that silencing of *PsRan* does not alter *Pst* virulence phenotype.

### Silencing of *PsRan* impedes fungal growth of *Pst*

**Figure 5:**
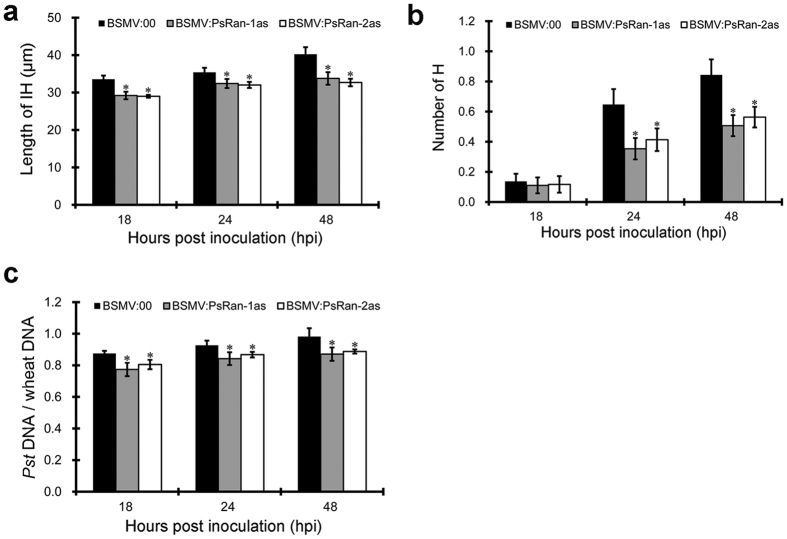
HIGS of *PsRan* impeded fungal growth of *Pst*. (**a**) The average length of infection hyphae (IH) per infection unit in control (BSMV:00-inoculated) and PsRan-silenced (BSMV:PsRan-1as- and BSMV:PsRan-2as-inoculated) wheat plants. The length of IH was measured from the substomatal vesicle to the apex of the longest infection hyphae. (**b**) The average number of haustoria (H) per infection unit at control and PsRan-silenced wheat plants. (**c**) Fungal biomass measurements using qRT-PCR analysis of total DNA extracted from control (BSMV:00-inoculated) and PsRan-silenced (BSMV:PsRan-1as- and BSMV:PsRan-2as-inoculated) wheat plants. Ratio of total *Pst* DNA to total wheat DNA was assessed using the *Pst* gene *PsEF1* and the wheat gene *TaEF1*. In (**a**–**c**), samples were taken at 18, 24, and 48 hpi with *Pst*, values represent mean ± standard errors of three biological replications (each done in triplicates), and differences were assessed using Student’s t-tests and asterisks indicate *P* < 0.05.

**Figure 6:**
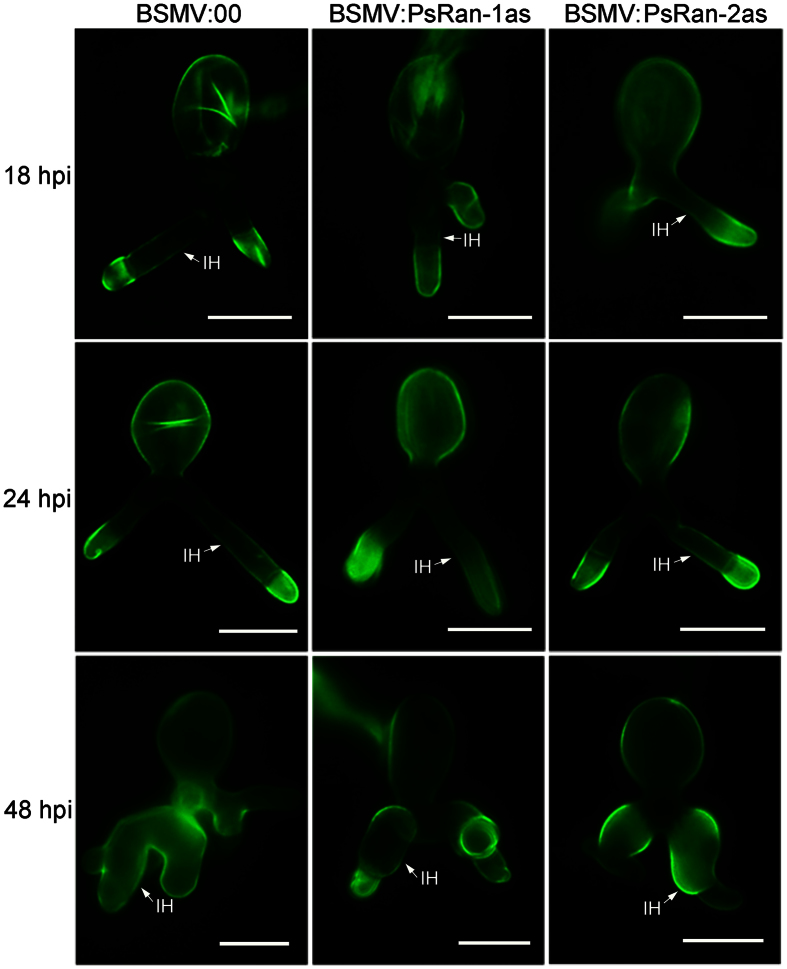
Micrographs of fungal growth in control (BSMV:00-inoculated) and PsRan-silenced (BSMV:PsRan-1as- and BSMV:PsRan-2as-inoculated) wheat plants. Infected wheat leaves were sampled at 18, 24, and 48 hpi with *Pst* and then were examined under an Olympus BX-53 microscope after staining with wheat germ agglutinin conjugated to the fluorophore Alexa-488. IH: infection hypha. Bar = 20 μm.

Despite of the unchanged virulence phenotype in PsRan-silenced wheat plants compared with control wheat plants (Fig. 4), we performed a cytological analysis of these wheat plants to investigate whether fungal growth of *Pst* is affected after silencing of *PsRan*. The number of haustoria and the length of infection hyphae were evaluated in PsRan-silenced and control wheat plants at 18, 24, and 48 hpi with *Pst*. Both the number of haustoria and the length of infection hyphae were significantly reduced in PsRan-silenced wheat plants compared with control wheat plants (Figs 5a,b and Fig. 6). In addition, qRT-PCR analysis showed that fungal biomass was also significantly decreased in PsRan-silenced wheat plants compared with control wheat plants (Fig. 5c), which is consistent with the cytological analysis. These results indicate that silencing of *PsRan* impedes fungal growth of *Pst*.

### Heterologous overexpression of *PsRan* in plant suppresses cell death triggered by a mouse BAX gene or a *Pst* Ras gene

**Figure 7:**
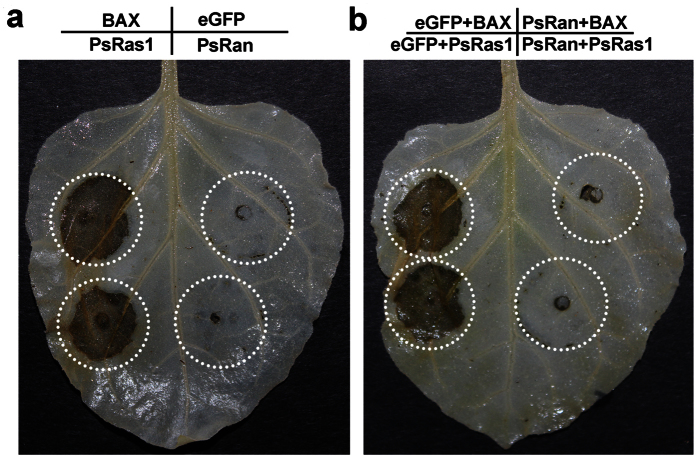
Herologous overexpression of *PsRan* in plant failed to induce cell death but suppressed cell death triggered by *BAX* or PsRas1. (**a**) Overexpression of *PsRan* and *eGFP* (negative control) could not trigger cell death in *N. benthamiana* as a mouse pro-apoptotic gene *BAX* and a *Pst* Ras gene *PsRas1* that can induce strong cell death in *N. benthamiana*. (**b**) Overexpression of *PsRan* in *N. benthamiana* suppressed cell death triggered by *BAX* and *PsRas1. N. benthamiana* leaves were infiltrated with *A. tumefaciens* cells containing PVX carrying *PsRan* or a control gene (*eGFP*), followed after 24 h by *A. tumefaciens* cells carrying PVX:BAX/PsRas1.

Because there are some similarities between fungal cell death and cell death in other higher eukaryotes^35^, we heterologously overexpressed *PsRan* in the model plant *N. benthamiana* to investigate the possible role of *PsRan* in cell death. Overexpression of *PsRan* in *N. benthamiana* did not trigger noticeable cell death as a mouse pro-apoptotic gene *BAX* or a *Pst* Ras gene *PsRas1* that can induce strong cell death in *N. benthamiana*^30^,^36^ (Fig. 7a). However, overexpression of *PsRan* could suppress the cell death triggered by BAX or PsRas1 (Fig. 7b). These results indicate that *PsRan* is involved in anti-cell death.

## Discussion

As molecular switches in cellular signal transduction pathways, Ran proteins have been shown to regulate a variety of important cellular processes in many eukaryotes. However, little is known about Ran function in pathogenic fungi. In the present study, we report the identification and functional analysis of a Ran gene from the important plant fungal pathogen *Pst*, which provides significant insight into Ran function in pathogenic fungi.

Only one Ran GTPase-encoding gene (*PsRan*) was identified in *Pst*. The same number of Ran-encoding gene is also observed in many other eukaryotes^1^. The PsRan protein contains all conserved domains of Ran GTPases and is localized to the nucleus as other known Ran proteins^33^,^34^, suggesting *PsRan* is a typical Ran gene. In addition, PsRan shares high (>70%) identity with other Ran proteins from fungi, plants, and animals. Previous studies showed that overexpression of various Ran proteins from plants, similarly to their mammalian/yeast homologues, suppressed the phenotype of the pim46-1 cell cycle mutant in yeast cells^37^,^38^. These observations indicate that Ran proteins in different organisms also have functional similarity besides conserved sequences.

qRT-PCR analysis showed that transcript level of *PsRan* was induced *in planta* during *Pst* infection, indicating *PsRan* may be important for plant infection of *Pst*. To investigate the specific role of *PsRan* in plant infection of *Pst*, we silenced *PsRan* using the BSMV-mediated HIGS system. The results showed that silencing of *PsRan* significantly decreased the number of *Pst* haustoria and the length of *Pst* infection hyphae, demonstrating the involvement of PsRan in fungal growth. Because little is currently known about Ran function in pathogenic fungi, this study is the first investigating of the role of Ran proteins from pathogenic fungi in fungal growth. In addition, other four families (Ras, Rho, Rab, and Sar1/Arf) in small GTP-binding proteins are also proven to regulate fungal growth^15^,^16^,^17^,^18^,^39^. These results highlight the great contribution of small GTP-binding proteins in fungal growth.

Successful *Pst* infection of wheat plants appears as a mass of uredinia arranged in long and narrow stripes on leaves^40^. However, PsRan-silenced wheat plants showed similar disease phenotypes as control wheat plants, with equivalent amounts of uredinia as control wheat plants, which indicate that silencing of *PsRan* did not alter *Pst* virulence phenotype. Silencing of many other rust genes from *Pst*, *Pt*, and *Pgt* also rarely changes their virulence phenotypes^41^,^42^. These results should not be taken as evidence that the majority of silenced genes are not involved in rust pathogenicity. The unchanged virulence phenotypes may be due to functional redundancy with other genes or that silencing was not sufficiently complete to knock down levels of encoded proteins to levels that would interfere with virulence phenotypes^42^.

Some small GTP-binding proteins are proven to be important regulators of cell death in different organisms^43^,^44^, and cell death is involved in several important biological processes in pathogenic fungi^35^. The possible roles of *PsRan* in cell death were investigated using heterologous systems because *Pst* lacks an efficient and reliable transformation system. Heterologous overexpression of *PsRan* in plant failed to induce cell death but suppressed cell death triggered by a mouse BAX gene or a *Pst* Ras gene, indicating that *PsRan* plays an important role in anti-cell death. Because there are some similarities between fungal cell death and cell death in other eukaryotes^35^, our results suggest that PsRan may truly function in the cell death of *Pst*. In addition, Ran proteins are also proven to be involved in human cell death, but as a death-promoting member^8^,^45^. These results indicate that Ran GTPases may function as positive or negative regulators of cell death in different organisms.

In conclusion, our study demonstrated that *PsRan* is involved in fungal growth and anti-cell death. Future studies should be directed toward the investigation of the specific mechanisms of *PsRan* in fungal growth and anti-cell death, which will contribute to the control of the wheat stripe rust disease.

## Methods

### Plant materials, strains and growth conditions

Wheat (*Triticum aestivum*) cv. Suwon 11, *N. benthamiana*, and five Chinese *Pst* isolates (CYR23, CYR29, CYR31, CYR32, and Su11-4) were used in this study. Wheat and *N. benthamiana* plants were grown at 20 °C and 25 °C, respectively. *Escherichia coli* JM109 was grown in a Luria-Bertani (LB) medium at 37 °C and used for plasmid construction. *A. tumefaciens* strain GV3101 was grown in LB medium at 28 °C and used for overexpression of *PsRan* in *N. benthamiana.* Antibiotics were used at final concentrations of 50 μg/ml ampicillin, 50 μg/ml kanamycin, 30 μg/ml rifampicin, and 25 μg/ml gentamycin.

### Sequence analysis and polymorphism analysis

The conserved domains of PsRan and other Ran proteins in different organisms were deduced using PFAM (http://pfam.xfam.org/). Sequence alignment between PsRan and other Ran proteins in different organisms was created using DNAMAN 6.0. Phylogenetic analysis of PsRan and other Ran proteins in different organisms was carried out with the MEGA5 software by the neighbor-joining method.

To identify intra-species polymorphism in *PsRan*, PCR amplifications were performed using cDNAs of the five Chinese *Pst* isolates (CYR23, CYR29, CYR31, CYR32, and Su11-4). The amplicons were then amplified, cloned, and sequenced. The re-sequenced genomes of the three US isolates (PST-21, PST-43, and PST-130) and the two UK isolates (PST-08-21 and PST-87-7) were used directly^46^. Local blast searches using BioEdit were conducted to identify the corresponding sequences, and DNAMAN6.0 was then used to create multiple sequence alignments. At each nucleotide position in the alignment, if there were different bases (one or more), one SNP was counted. The sum of this count was then calculated over all of the positions in each gene.

### Plasmid construction

The oligonucleotides used for plasmid construction in this study were documented in Supplementary Table S1. *PsRan* was cloned from the cDNA of *Pst* isolate CYR32 using FastPfu DNA Polymerase (TransGen Biotech, Beijing, China). To check the subcellular localization of *PsRan* in *N. benthamiana* and *S. pombe*, its ORF sequence was ligated into the plant binary expression vector pCAMBIA-1302 and the yeast expression vector pREP3X, respectively. To HIGS of *PsRan*, two specific partial cDNA regions were cloned into the BSMV gamma vector. For overexpression of *PsRan* in *N. benthamiana*, its ORF sequence was cloned into the PVX vector.

### Total RNA extraction and qRT-PCR

The total RNAs of urediniospores, germinated urediniospores and infected wheat leaves at different time points (18, 24, 48, 72, 120, 168, and 216 hpi) were isolated using TRIzol reagent (Invitrogen, Carlsbad, CA, USA). After urediniospores were incubated for 10 hours *in vitro*, germinated urediniospores were then harvested. A 2.0-μg RNA aliquot of each sample was used for cDNA synthesis with an oligo(dT)_18_ primer using the Reverse Transcription PCR system (Promega, Madison, WI, USA). Subsequently, SYBR green qRT-PCR assays were performed using a 7500 Real-Time PCR System (Applied Biosystems, Foster City, CA). The *Pst* housekeeping gene *EF1* was used as the endogenous reference to normalize the gene expression in *Pst*^47^. All reactions were performed in triplicate, and reactions without template were used as negative controls. The 2^−ΔΔCT^ method was used to quantify the relative gene expression levels^48^.

### Subcellular localization in *N. benthamiana*

The PsRan-GFP recombinant plasmid was introduced into the *Agrobacterium tumefaciens* strain GV3101 by electroporation. For infiltration of leaves, recombinant strains of *A. tumefaciens* were grown for 48 h, harvested, suspended in an infiltration media (10 mM MgCl_2_, 10 mM MES, pH 5.6, and 200 mM acetosyringone). Then *A. tumefaciens* suspensions were infiltrated at an OD_600_ of 0.8 into leaves of 4–6-week-old *N. benthamiana* plants using a syringe without a needle. Infiltrated plants were grown and maintained at 25 °C. Tissue samples were harvested at 2 or 3 d after infiltration and GFP signals were then observed at 2 dpi by an Olympus BX-53 microscope (Olympus Corporation, Tokyo, Japan) (excitation filter 485 nm, dichromic mirror 510 nm, and barrier filter 520 nm).

### Subcellular localization in *S. pombe*

The GFP sequence and PsRan-GFP fusion sequence that was gotten using the overlapping PCR method were cloned into the yeast expression vector pREP3X as previously described^49^. The two recombinant plasmids were transformed into *S. pombe* by electroporation and transformed cells were cultured as previously described^50^. The GFP signals of yeast cells were also observed by an Olympus BX-53 microscope (Olympus Corporation, Tokyo, Japan) (excitation filter 485 nm, dichromic mirror 510 nm, and barrier filter 520 nm).

### BSMV-mediated HIGS of *PsRan*

Capped *in vitro* transcripts were prepared from linearized plasmids containing the tripartite BSMV genome with the mMessage mMachine T7 *in vitro* transcription kit (Ambion, Austin, USA), following the manufacturer’s instructions. The second leaf of the wheat (Suwon 11) seedlings at the two-leaf stage was inoculated with BSMV transcripts by gently rubbing the surface with a gloved finger^51^. Three independent sets of plants were prepared for each of the four BSMV constructs (BSMV:00, BSMV:PsRan-1as, BSMV:PsRan-2as, and BSMV:TaPDS). The BSMV-infected plants were maintained in a growth chamber at 25 °C. Ten days after BSMV infection, the fourth leaves in BSMV:00-, BSMV:PsRan-1as- and BSMV:PsRan-2as-inoculated wheat plants were then inoculated with fresh virulent CYR32 urediniospores. The disease symptoms of the fourth leaves were recorded at 14 dpi with *Pst*. The inoculated fourth leaves were sampled at 18 hpi, 24 hpi, 48 hpi, and 14 dpi with *Pst* for RNA isolation to evaluate the silencing efficiencies of *PsRan* using qRT-PCR. These samples at 18, 24, and 48 hpi with *Pst* are also used for cytological observation of fungal growth and qRT-PCR analysis of fungal biomass.

### Uredinia quantification

The *Pst* disease phenotype was quantified by counting the number of uredinia within a 1-cm^2^ area at 14 dpi with *Pst*. To avoid bias among the leaf samples, leaves from at least five treated plants were randomly selected. Interpretation of the results was determined by comparing the values of the silenced plants to those of the controls.

### Cytological observation of *Pst* fungal growth in silenced wheat plants

Cytological analyses were performed to characterize *Pst* fungal growth in control and silenced wheat plants. Leaf segments (1.5 cm in length) cut from inoculated leaves were fixed and decolorized in ethanol/trichloromethane (3:1 v/v) containing 0.15% (w/v) trichloroacetic acid for 3–5 days. Decolorized leaf segments were stained with wheat germ agglutinin (WGA) conjugated to Alexa-488 (Invitrogen, Carlsbad, USA) as described previously^52^, and the stained samples were then examined under an Olympus BX-53 fluorescence microscope (Olympus Corporation, Tokyo, Japan) to observe *Pst* infection structures.

The number of haustoria and length of infection hyphae were measured as previously described^53^. For each wheat leaf sample in each biological replication, 30–50 infection sites from three leaves were examined to record number of haustoria and the length of infection hyphae per infection unit. The experiments were conducted in a completely randomized block design with three replications. Presence of a substomatal vesicle was defined as an established infection unit. The length of infection hyphae was measured from the substomatal vesicle to the apex of the longest infection hypha.

### Fungal biomass by qRT-PCR

To measure fungal biomass in infected wheat leaves, relative quantification of the single-copy target genes *PsEF1* (from *Pst*) and *TaEF1* (from wheat) by qRT-PCR was carried out as previously described^54^,^55^. Total genomic DNA of the wheat cv. Suwon 11 or the *Pst* isolate CYR32 was used to prepare standard curves derived from seven serial dilutions and the correlation coefficients for the analysis of the dilution curves were above 0.99. The relative quantities of the PCR products of the *Pst* gene *PsEF1* and the wheat gene *TaEF1* in infected wheat leaves were then calculated using the gene-specific standard curves to quantify the *Pst* and wheat genomic DNA, respectively.

### *A. tumefaciens*-mediated overexpression in *N. benthamiana*

The PVX:PsRan, PVX:eGFP, PVX:BAX, and PVX:PsRas1 recombinant plasmids were introduced into *A. tumefaciens* strain GV3101 by electroporation. Recombinant strains of *A. tumefaciens* were grown in LB liquid medium for 48 h, harvested, suspended in an infiltration medium (10 mM MgCl_2_) to an OD600 of 0.4.

To assay whether overexpression of *PsRan* in *N. benthamiana* can trigger cell death, *A. tumefaciens* suspensions carrying PVX:PsRan, PVX:eGFP (negative control), PVX:BAX (positive control), or PVX:PsRas1 (positive control) were infiltrated into the leaves of 4–6-week-old *N. benthamiana* plants using a syringe without a needle. The infiltrated *N. benthamiana* plants were grown and maintained in a cultivation room at 25 °C with a cycle of 16 h light/8 h darkness. Symptoms of plant cell death were monitored at 3 dpi with *A. tumefaciens*.

To assay suppression of BAX/PsRas1-triggered plant cell death, *A. tumefaciens* carrying PsRan were infiltrated initially, and then *A. tumefaciens* carrying BAX or PsRas1 were infiltrated into the same site 24 h later. Symptoms were monitored and photographed for 3 dpi with *A. tumefaciens* carrying BAX. *A. tumefaciens* cells carrying eGFP was infiltrated in parallel as controls.

## Additional Information

**How to cite this article**: Cheng, Y. *et al.* Characterization of a Ran gene from *Puccinia striiformis* f. sp. *tritici* involved in fungal growth and anti-cell death. *Sci. Rep.* **6**, 35248; doi: 10.1038/srep35248 (2016).

## Supplementary Material

Supplementary Information
