# Supplementary material for: Characterization of a Ran gene from Puccinia striiformis f. sp. tritici involved in fungal growth and anti-cell death
Source: Sci Rep. 2016 Oct 13;6:35248. doi: 10.1038/srep35248 (PMC5062253; doi:10.1038/srep35248)
Supplement: Supplementary Information [file srep35248-s1.pdf]

Characterization of a Ran gene from *Puccinia striiformis* f. sp. *tritici* involved in fungal growth and cell death

Yulin Cheng[1#], Juanni Yao[2#], Yanru Zhang[2], Shumin Li[2], Zhensheng Kang[2]*

[1]State Key Laboratory of Crop Stress Biology for Arid Areas, College of Life Sciences, Northwest A&F University, Yangling 712100, Shaanxi, China

[2]State Key Laboratory of Crop Stress Biology for Arid Areas, College of Plant Protection, Northwest A&F University, Yangling 712100, Shaanxi, China

[#]These authors contributed equally to this work.

*To whom correspondence should be addressed. E-mail: kangzs@nwsuaf.edu.cn

Target	Score (Bits)	Expect	Alignment Length	Identities	Positives	
P. triticina 1-1 BBBD Race 1 (V2): PTTG_02103.4: GTP-binding nuclear protein spi1	331.257	0.0	184	156	168	Ran proteins
P. graminis tritici: PGTG_04433.4: GTP-binding nuclear protein spi1	328.946	0.0	184	155	167	Ran proteins
P. striiformis PST-78: PSTG_13752.1: GTP-binding nuclear protein spi1	328.946	0.0	184	156	166	Ran proteins
P. graminis tritici: PGTG_17558.4: GTP-binding protein ypt1	110.538	1.03353E-24	161	58	91	Rab proteins
P. triticina 1-1 BBBD Race 1 (V2): PTTG_05460.4: GTP-binding protein ypt1	110.538	1.10487E-24	161	58	91	Rab proteins
P. triticina 1-1 BBBD Race 1 (V2): PTTG_10008.4: GTP-binding protein ypt5	109.768	1.54262E-24	175	62	98	Rab proteins
P. graminis tritici: PGTG_09844.4: rab family protein	108.612	3.55322E-24	173	61	97	Rab proteins
P. striiformis PST-78: PSTG_11722.1: GTP-binding protein ypt5	108.612	3.83032E-24	162	58	94	Rab proteins
P. graminis tritici: PGTG_15757.4: rab family protein	102.064	3.86465E-22	182	56	97	Rab proteins
P. triticina 1-1 BBBD Race 1 (V2): PTTG_04650.4: GTP-binding protein ryh1	101.293	5.53258E-22	182	56	97	Rab proteins
P. graminis tritici: PGTG_00987.4: Ras-like protein Rab-8A	99.7525	1.59636E-21	161	51	88	Rab proteins
P. striiformis PST-78: PSTG_12637.1: GTP-binding protein ryh1	99.3673	2.03337E-21	185	58	100	Rab proteins
P. striiformis PST-78: PSTG_11158.1: Ras-like protein Rab-8A	99.3673	2.48418E-21	161	50	88	Rab proteins
P. triticina 1-1 BBBD Race 1 (V2): PTTG_07767.4: GTP-binding protein ypt3	98.9821	2.70035E-21	154	54	82	Rab proteins

Figure S1. A BLAST search using the know Ran protein GSP1 from *S. cerevisiae* as the query in the Broad Institute *Puccinia* database. *PSTG_13752.1* indicates *PsRan*.

Target	Score (Bits)	Expect	Alignment Length	Identities	Positives
P. triticina 1-1 BBBD Race 1 (V2): PTTG_02103.4: GTP-binding nuclear protein spi1	345.51	0.0	195	161	175
P. graminis tritici: PGTG_04433.4: GTP-binding nuclear protein spi1	343.584	0.0	195	160	174
P. striiformis PST-78: PSTG_13752.1: GTP-binding nuclear protein spi1	343.584	0.0	195	161	173
P. graminis tritici: PGTG_17558.4: GTP-binding protein ypt1	110.153	1.16146E-24	161	57	91
P. triticina 1-1 BBBD Race 1 (V2): PTTG_05460.4: GTP-binding protein ypt1	109.768	1.76274E-24	161	57	91
P. graminis tritici: PGTG_09844.4: rab family protein	107.457	7.91486E-24	162	58	94
P. triticina 1-1 BBBD Race 1 (V2): PTTG_10008.4: GTP-binding protein ypt5	107.457	8.74836E-24	162	58	94
P. striiformis PST-78: PSTG_11722.1: GTP-binding protein ypt5	107.457	9.04527E-24	162	58	94
P. graminis tritici: PGTG_15757.4: rab family protein	100.523	9.20281E-22	183	57	97
P. triticina 1-1 BBBD Race 1 (V2): PTTG_04650.4: GTP-binding protein ryh1	100.523	9.51514E-22	183	57	97
P. graminis tritici: PGTG_00987.4: Ras-like protein Rab-8A	99.7525	1.87038E-21	161	51	88
P. striiformis PST-78: PSTG_12637.1: GTP-binding protein ryh1	99.3673	2.44279E-21	183	56	98
P. striiformis PST-78: PSTG_11158.1: Ras-like protein Rab-8A	98.5969	3.73846E-21	161	50	88
P. triticina 1-1 BBBD Race 1 (V2): PTTG_05367.4: Ras-like protein Rab-8A	98.5969	3.96331E-21	161	50	88
P. triticina 1-1 BBBD Race 1 (V2): PTTG_07767.4: GTP-binding protein ypt3	98.2117	4.84201E-21	154	54	82

⇨ Ran proteins

⇨ Rab proteins

Figure S2. A BLAST search using the know Ran protein GSP2 from *S. cerevisiae* as the query in the Broad Institute *Puccinia* database. *PSTG_13752.1* indicates *PsRan*.

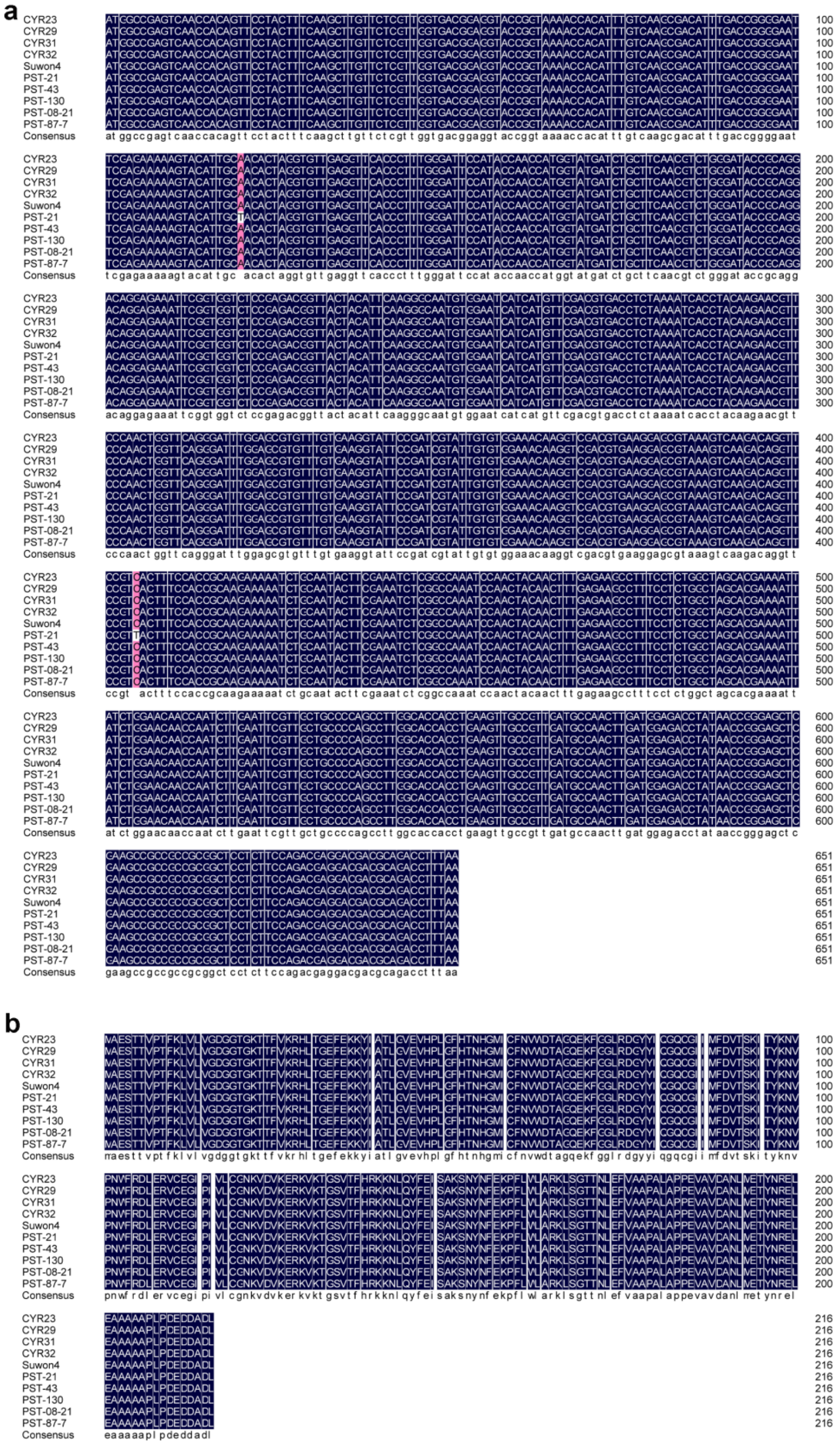

Figure S3. Sequence alignment of *PsRan* among different *Pst* isolates in the nucleotide level (a) and amino acid level (b). Ten *Pst* isolates were used, including five Chinese isolates (CYR23, CYR29, CYR31, CYR32, and Su11-4), three US isolates (PST-21, PST-43, and PST-130) and two UK isolates (PST-08-21 and PST-87-7).

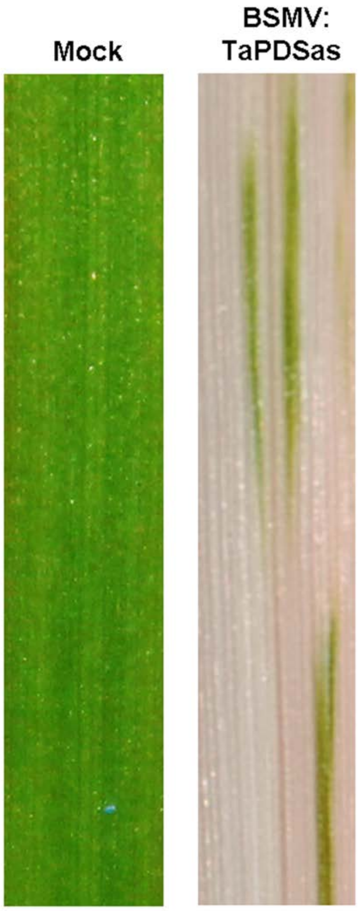

Figure S4. Phenotypes of wheat plants 10 dpi with FES buffer (Mock) and BSMV:TaPDS, respectively.

Table S1 Primers used in this study.

Primer name	Primer 5'-3'	Purpose
PsEF1-qRT-F	TTCGCCGTCCGTGATATGAGACAA	The endogenous reference for normalization in qRT-PCR
PsEF1-qRT-R	ATGCGTATCATGGTGGTGGAGTGA	The endogenous reference for normalization in qRT-PCR
PsRan-qRT-F	ATTTGGAGCGTGTTTGTGAA	qRT-PCR for validation of the expression patterns of PsRan
PsRan-qRT-R	GACGGAACCTGTCTTGACTTTA	qRT-PCR for validation of the expression patterns of PsRan
PsRan-PacI-F1	CCTTAATTAAGACCCAGATTCCGTTTACG	Clone PsRan-1as to the r vector for HIGS
PsRan-NotI-R1	TATGCGGCCGCTACCGGTACCTCCGTCAC	Clone PsRan-1as to the r vector for HIGS
PsRan-PacI-F2	CCTTAATTAAGCCTTGGCACCACCTGA	Clone PsRan-2as to the r vector for HIGS
PsRan-NotI-R2	TATGCGGCCGCGTCTGCGTCGTCCTCGTCT	Clone PsRan-2as to the r vector for HIGS
PsRan-SpeI-F	GACTAGTATGGCCGAGTCAACCACAGT	Clone PsRan to pCAMBIA-1302 for expression in *N. benthamiana*
PsRan-AvrII-R	TTCCTAGGAAGGTCTGCGTCGTCCTCG	Clone PsRan to pCAMBIA-1302 for expression in *N. benthamiana*
PsRan-ClaI-F	GGCATCGATATGGCCGAGTCAACCACAGT	Clone PsRan to PVX for expression in *N. benthamiana*
PsRan-SmaI-R	GTACCCGGGTTAAAGGTCTGCGTCGTCCTC	Clone PsRan to PVX for expression in *N. benthamiana*
GFP-XhoI-F	CCGCTCGAGATGGTGAGCAAGGGCGA	Clone GFP to pREP3X for expression in *S. pombe*
GFP-BamHI-R	GCGGATCCCTTGTACAGCTCGTCCATGC	Clone GFP to pREP3X for expression in *S. pombe*
Fusion-XhoI-F1	CCGCTCGAGATGGTGAGCAAGGGCGA	Overlapping PCR
Fusion-R1	GGTTGACTCGGCCATCTTGTACAGCTCGTC	Overlapping PCR
Fusion-F2	GACGAGCTGTACAAGATGGCCGAGTCAACC	Overlapping PCR
Fusion-BamHI-R2	GCGGATCCTTAAAGGTCTGCGTCGTCCT	Overlapping PCR
TaEF1-qRT-F	TGGTGTCATCAAGCCTGGTATGGT	Calculation of fungal biomass
TaEF1-qRT-R	ACTCATGGTGCATCTCAACGGACT	Calculation of fungal biomass
